# Green Synthesis of Carbon Quantum Dots and Carbon Quantum Dot-Gold Nanoparticles for Applications in Bacterial Imaging and Catalytic Reduction of Aromatic Nitro Compounds

**DOI:** 10.1021/acsomega.4c00833

**Published:** 2024-04-24

**Authors:** Xuan-Wei Fang, Hao Chang, Tsunghsueh Wu, Chen-Hao Yeh, Fu-Li Hsiao, Tsung-Shine Ko, Chiu-Lan Hsieh, Mei-Yao Wu, Yang-Wei Lin

**Affiliations:** † Department of Chemistry, National Changhua University of Education, 1 Jin-De Road Changhua City 50007, Taiwan; ‡ Department of Chemistry, University of Wisconsin-Platteville, 1 University Plaza Platteville Wisconsin 53818-3099, United States; § Department of Materials Science and Engineering, Feng Chia University, 100, Wenhwa Road Taichung City 40724, Taiwan; ∥ Graduate Institute of Photonics, National Changhua University of Education, 1 Jin-De Road Changhua City 50007, Taiwan; ⊥ Department of Electronic Engineering, National Changhua University of Education, 1 Jin-De Road Changhua City 50007, Taiwan; # Department of Biology, National Changhua University of Education, 1 Jin-De Road Changhua City 50007, Taiwan; ∇ School of Post-baccalaureate Chinese Medicine, China Medical University, 91, Hsueh-Shih Road Taichung 40424, Taiwan

## Abstract

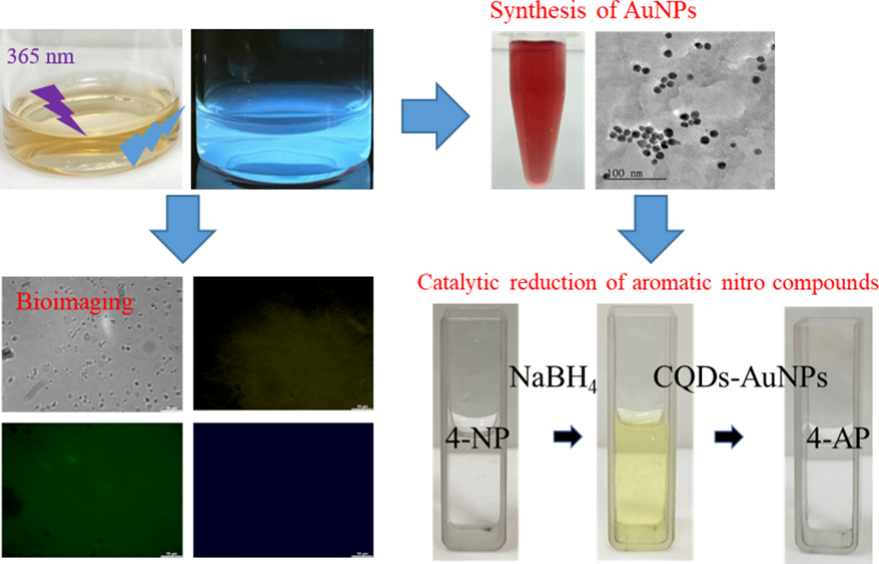

This study delves into the green synthesis and multifaceted applications of three types of carbon quantum dots (CQDs), namely, CQDs-1, CQDs-2, and CQDs-3. These CQDs were innovatively produced through a gentle pyrolysis process from distinct plant-based precursors: genipin with glucose for CQDs-1, genipin with extracted gardenia seeds for CQDs-2, and genipin with whole gardenia seeds for CQDs-3. Advanced analytical techniques, including X-ray photoelectron spectroscopy (XPS) and Fourier-transform infrared spectroscopy (FT-IR), were employed to detail the CQDs’ structural and surface characteristics, revealing their unique functional groups and surface chemistries. The study further explores the CQDs’ bioimaging potential, where confocal fluorescence microscopy evidenced their swift uptake by *Escherichia coli* bacteria, indicating their suitability for bacterial imaging. These CQDs were also applied in the synthesis of gold nanoparticles (AuNPs), acting as reducing agents and stabilizers. Among these, CQD3-AuNPs were distinguished by their remarkable stability and catalytic efficiency, achieving a 99.7% reduction of 4-nitrophenol to 4-aminophenol in just 10 min and maintaining near-complete reduction efficiency (99.6%) after 60 days. This performance notably surpasses that of AuNPs synthesized using sodium citrate, underscoring the exceptional capabilities of CQD3-AuNPs. These insights pave the way for leveraging CQDs and CQD-stabilized AuNPs in bacterial imaging and catalysis, presenting valuable directions for future scientific inquiry and practical applications.

## Highlights

- Three unique CQDs were synthesized through a single-step mild pyrolysis process.
- CQDs-3 exhibit maximum quantum yield of 4.0%.
- Rapid internalization of CQDs into *E. coli* was confirmed through vivid three-color confocal fluorescence microscopy.
- CQDs are used as both reducing agents and stabilizers in the synthesis of AuNPs.
- CQD3-AuNPs reduce 99.7% of 4-NP to 4-AP within 10 min, maintaining 99.6% reduction efficiency after 60 days.

## 1 Introduction

Carbon quantum dots (CQDs) represent a novel class of nanomaterials with sizes typically below 10 nm, featuring distinctive optical properties.^1−3^ Their optical responses, uniquely influenced by the wavelength of the excitation light source, produce fluorescence emissions, rendering their wide applications in sensors, catalysis, and bioimaging.^4−6^ As bioimaging is the key to advancing biomedical field, materials for bioimaging have been at the forefront of this burgeoning field, and their material properties, such as biocompatibility, size, surface functional group, fluorescence intensity, and chemical stability, have significant influence on their acceptance to the field.^7^ CQDs exhibit excellent biocompatibility, ideal for bioanalysis in cells and tissues. One key advantage of CQDs over other nanomaterials is that their carbon surface can be modified to create rich functional groups on the surface of CQDs enabling reactions and functional modifications with specific targeted species in bioanalysis.^8^

Common methods for synthesizing CQDs include electrochemical methods, hydrothermal methods, microwave-assisted synthesis, ultrasound-assisted synthesis, oxidation methods, and reduction methods.^9^ In recent years, studies have identified gardenia seeds as a viable carbon source for synthesizing CQDs.^10,11^ The gardenia seeds are rich in functional groups on their surface, and deriving CQDs from plant seeds aligns with the principles of waste utilization, offering a sustainable, environmentally friendly, low-toxicity, and green synthesis approach for CQDs.^12,13^ However, some drawbacks exist in these methods such as stringent synthesis conditions and relatively long reaction times. Researchers have explored the use of pyrolysis as an alternative method for CQDs synthesis.^10,14^ Pyrolysis presents the advantages of simplicity and ease of operation, requiring no expensive instruments or chemicals, making it cost-effective. CQDs synthesized through pyrolysis exhibit excellent biocompatibility, optical stability, and high quantum yields, leading to widespread applications in fields such as biodetection, cell imaging, and biomedicine.^7^ In comparison to other synthesis methods, pyrolysis demonstrated significant advantages. Traditional approaches like hydrothermal methods demand high-temperature and high-pressure conditions with prolonged reaction times and lower yields. Therefore, pyrolysis stands out as an improved method without these drawbacks in CQDs synthesis.

Nitrophenol derivatives have posed significant environmental issues due to their widespread applications in the manufacturing of pesticides, explosives, pharmaceuticals, and dyes and playing a crucial role in the bleaching process of the paper industry.^15^ Moreover, nitrophenol derivatives are extensively used as herbicides, insecticides, and fungicides in common large-scale agriculture practices.^16^ In addition to human activities for the release of nitrophenol derivatives to the environment, the other cause of the prevalence of these compounds in nature is the natural decomposition of leaves and woody materials leaching to soil, sediments, surface water, and groundwater.^17,18^ Due to the high toxicity of phenol derivatives and their long-term accumulation in the environment, some of these compounds have been prioritized as pollutants by the U.S. Environmental Protection Agency. Among them, 4-nitrophenol (4-NP) poses significant health risks, including pancreatic and liver damage, hypertension, protein denaturation, irritations to the eyes and skin, and anemia.

To mitigate the presence of 4-NP in wastewater, various methods have been developed, including photocatalytic decomposition, electrochemical treatment, chemical precipitation, and membrane separation.^19−21^ Among these different separation methods, researchers have increasingly favored in situ catalytic reduction, converting 4-NP to 4-aminophenol (4-AP). This method is considered to be more economically viable, environmentally friendly, and efficient. Besides lowering the toxicity, such conversion can create 4-AP, which is an important intermediate for the preparation of corrosion inhibitors, photographic developers, and analgesic and antipyretic drugs. In recent years, extensive research has indicated that catalyzing the direct hydrogenation of 4-NP using metal nanoparticles is recognized as the primary method for producing 4-AP.^22−24^ Therefore, developing safe, cost-effective, and environmentally friendly metal nanoparticles presents a challenge for catalyzing 4-NP. Among the known studies, several types of metal nanoparticles, such as Ni, Ag, and Cu, have been employed for catalyzing the reduction of 4-NP.^25−27^ Gold nanoparticles (AuNPs) are considered one of the most promising catalysts due to their chemical stability.

This study chose CQDs as a reducing agent for synthesizing AuNPs. The CQDs were derived from genipin (GNP) and gardenia seeds. These carbon sources offer natural, easily accessible, cost-effective, and waste-utilizing advantages, providing significant benefits for CQD synthesis. In this study, we employed a pyrolysis method to synthesize CQDs, aiming to overcome the drawbacks of the hydrothermal method and allow for large-scale production with minimum waste generation. Furthermore, this improvement significantly reduced the required synthesis time, simplified the operation, and eliminated the need for expensive instruments. Due to the characteristics of CQDs, we also applied them in bacterial imaging with the expectation of extending their use to the field of biomedical imaging in the future. Furthermore, in this study, AuNPs were synthesized by seed-derived CQDs by using a one-pot method. In this process, there is no need for additional stabilizers, allowing the synthesis of highly stable CQDs-AuNPs without additional chemicals, aligning with the principles of green chemistry. Finally, in addition to catalyzing the reduction of 4-NP with newly developed CQDs-AuNPs, this study demonstrated success in the reduction of various other aromatic nitro compounds, aiming to remediate toxic materials to less toxic products.

## 2 Materials and Methods

### 2.1 Chemicals

All reagents utilized in this study were procured from Sigma-Aldrich (Milwaukee, WI, USA). Deionized water with a resistivity of 18.2 MΩ·cm^–1^, obtained from a Milli-Q ultrapure system, was employed throughout the experimental procedures. Gardenia seeds, both those that had undergone extraction and those in their natural state, played a pivotal role in the synthesis process and were graciously provided by Chiu-Lan Hsieh from the National Changhua University of Education, Changhua.

### 2.2 Characterization

The synthesized CQDs and CQDs-AuNPs were characterized using various analytical techniques. UV–visible spectra were collected using an Evolution 200 UV–vis spectrophotometer (ThermoFisher, NY, USA) to assess the optical properties. The crystal structures were examined through X-ray diffraction (XRD) patterns obtained via a LabX XRD-6000 X-ray diffractometer (SHIMADZU, Kyoto, Japan). Fourier transform infrared (FT-IR) spectroscopy on an Agilent Cary 600 instrument (Agilent, CA, USA) identified organic functional groups. Morphology and microstructure analyses were conducted using high-resolution transmission electron microscopy (HRTEM) on a JEOL-1200EX II TEM (JEOL, Tokyo, Japan). Photoluminescence (PL) spectra, revealing emission properties, were acquired using a Synergy H1 Hybrid Multimode Microplate Reader (Biotek Instruments, Inc., Winooski, VT, USA). X-ray photoelectron spectroscopy (XPS) on a VG ESCA210 instrument (VG ESCA210; VG Scientific, West Sussex, UK) validated the surface status. Finally, the potential bioimaging applications were explored through cell imaging by using a Carl Zeiss 510 LSM laser scanning confocal microscope. This comprehensive characterization provides a detailed understanding of the structural, optical, and surface properties of the synthesized materials.

### 2.3 Preparation of CQDs

**Scheme 1:**
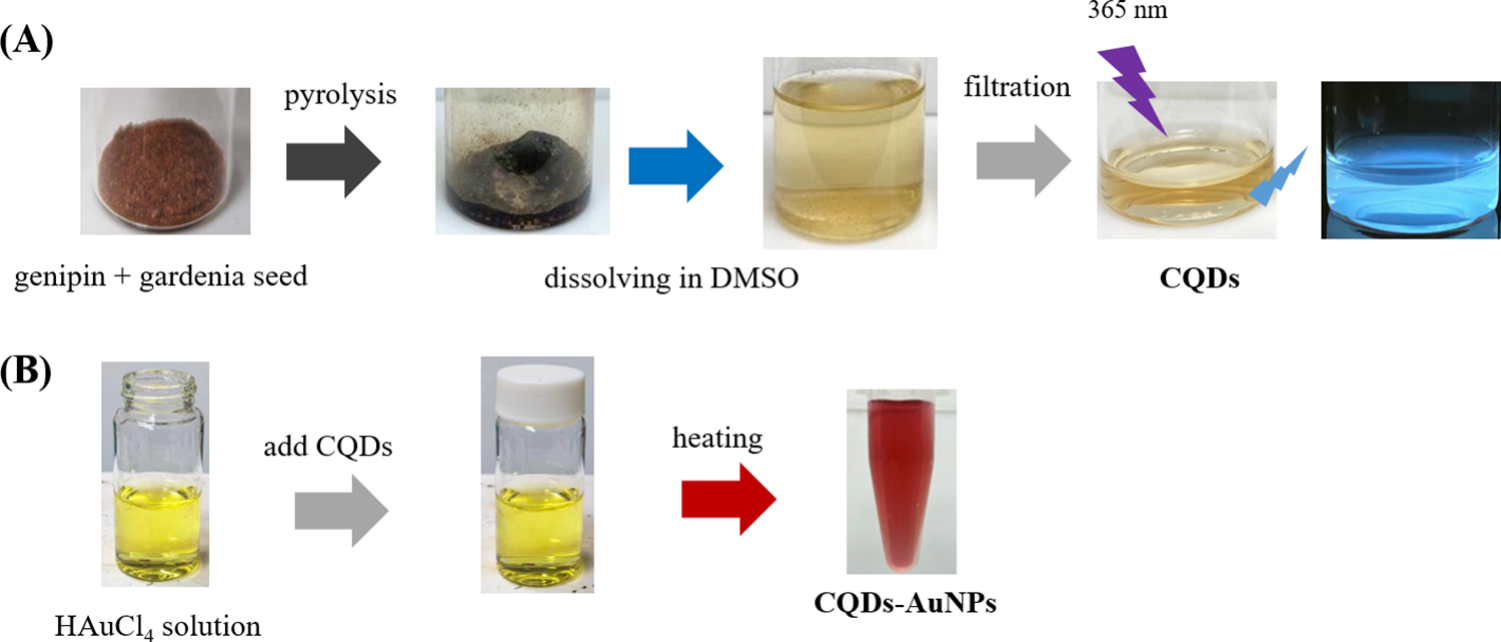
Schematic Procedure for Green Synthesis of (A) Luminescent CQDs through a One-Step Mild Pyrolysis Process and (B) AuNPs Using CQDs as the Reducing Agent and Stabilizer Photography courtesy of Xuan-Wei Fang. Copyright 2024.

In this study, a systematic synthesis approach involving the combination of GNP with distinct plant extracts was employed to produce CQDs. Three discrete systems, denoted as CQDs-1, CQDs-2, and CQDs-3, were meticulously produced. The preparation of CQDs-1 involved the precise combination of 50 mg of GNP with an equal amount of glucose. For CQDs-2, the formulation included 50 mg of GNP and 50 mg of ground gardenia seeds that had undergone extraction (extracting GNP by ethanol). In the case of CQDs-3, the combination comprised 50 mg of GNP and 50 mg of ground gardenia seeds. Following careful sealing in 25 mL vials, the mixtures were heated in an oven at 220 °C for 2 h to yield dark black residues. These residues were then cooled to room temperature (∼25 °C) and dissolved in 10 mL of DMSO. Efficient dissolution was achieved through stirring and ultrasonic vibration (DC200H, Honeywell, Charlotte, NC, USA). Following a 24 h dark incubation period, the samples underwent three successive rounds of ultrasonic vibration and filtration using a 0.22 μm syringe filter, ultimately yielding the desired CQDs (Scheme 1A).

### 2.4 Preparation of CQDs-AuNP Composites

A 10 mL portion of 1 mM HAuCl_4_ solution was dispensed into a small glass bottle. Then, 8 mL of deionized water and 2 mL of the previously prepared CQDs were added into the bottle. The bottle was sealed with plastic wrap, creating small punctures on the plastic wrap with a needle. Heat was applied to the mixture under continuous stirring until it boiled. After being boiled for 1 min, the wine-red color solution was formed, confirming the successful one-pot synthesis of AuNPs (Scheme 1B). In this study, AuNPs were identified as CQD1-AuNPs, CQD2-AuNPs, and CQD3-AuNPs, when they were synthesized using CQDs-1, CQDs-2, and CQDs-3, respectively.

### 2.5 Antibacterial Evaluation and Bacterial-CQD Conjugation

A solitary colony of *E. coli* strain BRBC 12438 was selected from solidified agar plates and inoculated into 1.0 mL of Luria–Bertani (LB) medium. The bacterial cultures were then incubated at 37 °C with continuous shaking at 200 rpm until the optical density at 600 nm wavelength reached 1.0, measured with an optical path length of 1.0 cm. Subsequently, each bacterial suspension underwent centrifugation at 3000*g* for 10 min at 25 °C, and the resulting bacterial pellets were washed three times with phosphate-buffered saline (PBS).

To evaluate the antibacterial activity of the synthesized CQDs against *E. coli*, a suspension containing 5.0 × 10^3^ colony-forming units (CFU) per milliliter was evenly spread on solidified LB agar plates. Six paper ingots were subsequently placed on the agar plate surface, and different concentrations of CQDs (ranging from 0 to 32 mg/mL) were applied to the paper ingots. A positive control group utilized a concentration of 32 mg/mL of benzoic acid (BA). After a 24 h incubation period, the size of the inhibition zone around the paper ingots was measured and compared among the various treatment groups.

For the formation of bacterial-CQDs conjugates, the bacterial cells were treated with a 70% (v/v) ethanol solution to enhance the internalization of the CQDs. This step was carried out at 4 °C for 5 min. The cells were then stained by dispersing them in a 100 mM phosphate buffer containing CQDs at a concentration of 25.6 mg/mL for 10 min at room temperature. After staining, the cell-CQD conjugates were thoroughly washed with DI water for three wash cycles. Finally, 20 mL of the resulting conjugate solution was transferred to a glass slide for the fluorescence measurements. Fluorescence images of the cells were captured by using laser excitations at wavelengths of 365, 475, and 532 nm.

### 2.6 NaBH_4_ Reduction of 4-NP in the Presence of CQDs-AuNPs

A quantity of 20 μL of 4-NP solution (10 mM) was extracted using a micropipette and introduced into the quartz cell. Subsequently, the mixture is supplemented with 800 μL of 100 mM NaBH_4_ to initiate the catalytic reduction of the 4-NP solution. To confirm the pivotal role of CQDs-AuNPs as the primary catalyst in the reduction reaction, UV–visible spectroscopy was employed to monitor the characteristic absorption peak of 4-NP at 400 nm within the initial minute. At the 60 s mark, we introduced 200 μL of CQDs-AuNPs and continued the monitoring process for 10 min to observe alterations in the characteristic peak absorption at 400 nm. This meticulously designed procedure aims to systematically evaluate the catalytic reduction efficiency of CQDs-AuNPs.

## 3 Results and Discussion

### 3.1 Green Synthesis and Characterization of CQDs

The X-ray diffraction (XRD) analysis of CQDs, as illustrated in Figure S1A, revealed a pronounced broad reflection peak at 2θ = 22°. This peak is attributed to the (002) crystal plane, indicative of the graphite-like structure characteristic of the CQDs. This finding aligns with the structural properties observed in numerous other carbon dot studies, confirming the graphitic nature of our CQDs.^28−31^ The surface functional groups of CQDs were identified using FT-IR analysis (Figure S1B). The broad absorption band at 3335 cm^–1^ was attributed to −OH stretching vibration. Specific stretching vibration of C=O was observed at 1679 cm^–1^, while the band at 1442 cm^–1^ indicated C=C stretching vibrations, suggesting the carbonization of organic compounds into graphite-like nanostructures. These results provide strong evidence of the functionalization of CQDs with hydroxyl, carbonyl, and carboxylic acid groups. These surface functional groups offer valuable insights into the PL mechanisms of CQDs, making them suitable and environmentally friendly probes for biochemical research. Transmission electron microscopy (TEM) images of CQDs (Figure S1C) revealed well-distributed spherical CQDs-1, CQDs-2, and CQDs-3 with an average diameter of approximately 2.1 ± 1.8, 0.6 ± 0.5, and 0.7 ± 0.3 nm, respectively. The observed smaller particle sizes in CQDs-2 and CQDs-3 than in CQDs-1 suggest that the extracted gardenia seed contains abundant functional groups and the pyrolysis process allows the precursor materials to decompose more completely, generating smaller organic fragments. These thermally decomposed organic fragments undergo a series of assembly processes, resulting in the formation of smaller carbon dots.

**Figure 1:**
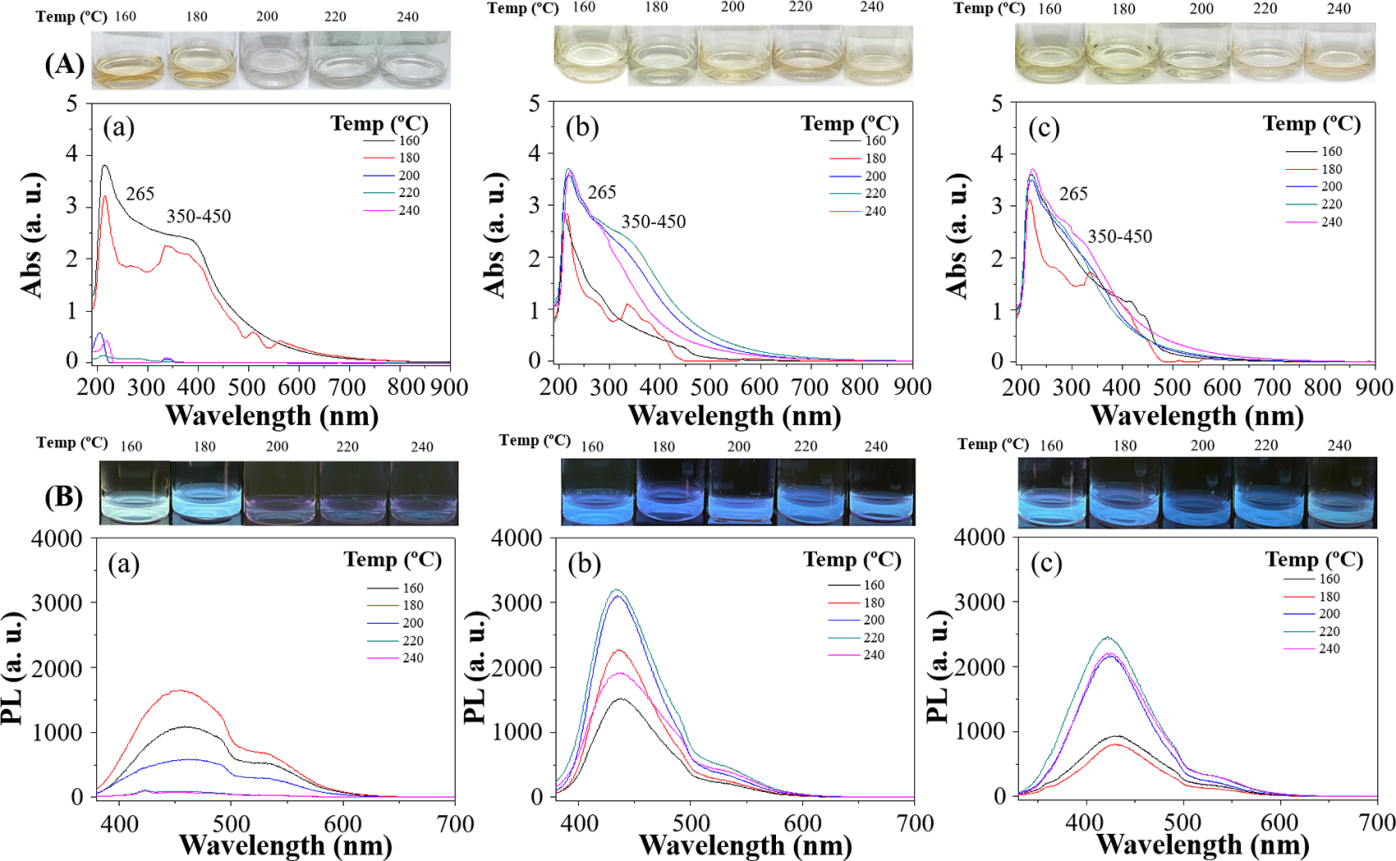
(A) UV–vis spectra and (B) photoluminescence of (a) CQDs-1 (50 mg GNP + 50 mg glucose), (b) CQDs-2 (50 mg GNP + 50 mg extracted gardenia seed), and (c) CQDs-3 (50 mg GNP + 50 mg gardenia seed) prepared at different temperatures (160, 180, 200, 220, and 240 °C). Photographic images of CQDs synthesized at different temperatures (160, 180, 200, 220, and 240 °C) under daylight conditions and their corresponding fluorescence properties when excited with UV light at a wavelength of 365 nm (photography courtesy of Xuan-Wei Fang. Copyright 2024).

In the search for optimum pyrolysis temperature, the optical properties of all CQDs were characterized with UV–vis spectroscopy after the pyrolysis. Suitable CQDs for cell imaging should display distinct stable absorption and fluorescence properties. Characteristic peaks of promising CQDs showed the absorption peak at 265 nm of excitation wavelength, indicating π → π* electronic transitions associated with the conjugated double bonds (C=C) present on CQDs. Additionally, another absorption peak at 400 nm was identified, attributed to n → π* transitions associated with C=O groups or C–OH in sp3 hybridized bonds present on CQDs (as shown in Figure 1). For CQDs-1, caramelization occurred at a pyrolysis temperature over 180 °C, leading to a low yield of CQDs, which was confirmed by low absorption signals from the UV–vis spectrum (Figure 1Aa). The cause can be attributed to the complete carbonization of glucose at temperatures surpassing 180 °C, impeding the liberation of CQDs. The black carbons were settled to the bottom of the solution, and the solution became transparent and colorless. In contrast, CQDs-2 and CQDs-3, formulated with GNP and gardenia seeds, exhibited greater tolerance to elevated reaction temperatures (Figure 1Ab,c), resulting in light amber color solutions. Our study showed that complete carbonization occurred at temperatures over 240 °C (not shown in Figure 1) unfavorable for CQD formation. The optimum processing temperature should also be decided after fluorescence measurements.

All three CQDs were characterized for their fluorescence properties and unique shifts in PL wavelength from different processing temperatures. CQDs with UV absorption produced a fluorescence band at about 445 nm from 365 nm excitation (Figure 1B). The PL signal intensity increases with rising reaction temperatures; however, excessive temperatures lead to severe carbonization, resulting in a sharp decrease in PL. The highest permissible temperature for CQDs-1 is 180 °C and 220 °C for CQDs-2 and CQDs-3. Furthermore, elevating the reaction temperature causes the CQDs to inherit a slight blue shift in the PL wavelength, attributed to the different compositions of CQDs formed at varying reaction temperatures. Using quinine sulfate as the standard in 0.1 M H_2_SO_4_ (with a quantum yield of 54%), the quantum yields of CQDs, are summarized in Table 1. Notably, CQDs-3 achieves the highest quantum yield of 4.0% at a reaction temperature of 220 °C. Therefore, 220 °C is chosen as the optimal reaction temperature for CQD synthesis. The PL characteristics of CQDs prepared at 220 °C were further examined at various excitation wavelengths ranging from 300 to 420 nm (Figure S2). The PL spectrum of CQDs exhibits a bathochromic shift, with a gradual red shift toward longer wavelengths accompanied by a decrease in PL intensity. The prepared CQDs exhibit a broad distribution in size, showcasing diverse optical properties on the nanoscale. Consequently, under excitation at different wavelengths, irradiation of CQDs of varying sizes leads to the phenomenon of redshift in the PL spectrum.

**Table 1:** Quantum Yield (%) of CQDs Prepared at Different Temperatures

**temp. (°C)**	**160**	**180**	**200**	**220**	**240**
CQDs-1^a^	0.31	0.34	0.01	d	d
CQDs-2^b^	0.27	0.34	0.47	0.51	0.30
CQDs-3^c^	1.97	1.71	2.17	4.00	2.00

a CQDs-1:50 mg genipin (GNP) + 50 mg glucose.

b CQDs-2:50 mg GNP + 50 mg extracted gardenia seeds.

c CQDs-3:50 mg GNP + 50 mg gardenia seeds.

d ND.

A systematic study was conducted on three synthesis approaches (CQDs-1, CQDs-2, and CQDs-3) to examine the effects of precursor weight ratios on PL quantum yield. The total weight of precursors was maintained at 100 mg, while different mixing ratios (mg) of 10:90, 30:70, 50:50, 70:30, and 90:10, were examined. From Table 2, the results indicated that both CQDs-1 and CQDs-2 synthesis routes exhibited relatively lower quantum yields with insignificant differences. However, the CQDs-3 system achieved the highest quantum yield of 4.0% at a 50 mg:50 mg mixing ratio. The study also found that when the GNP amount was lower than gardenia seed, the quantum yield decreased, highlighting the substantial role of GNP in enhancing the quantum yield. Conversely, when the GNP amount exceeded the gardenia seed, the quantum yields also declined, possibly due to the insufficient protective surface functional groups provided by the gardenia seed, leading to a reduction in quantum yield.^5^ Therefore, considering the influence of different mixing amounts on quantum yield, the study selected the 50 mg:50 mg mixing amounts as the optimized weight ratio between GNP and gardenia seeds for the three synthetic approaches.

**Table 2:** Quantum Yield (%) of CQDs Prepared at 220°C with Different Weight Compositions

**weight composition (mg)**	**10:90**	**30:70**	**50:50**	**70:30**	**90:10**
CQDs-1^a^	d	d	d	0.14	0.17
CQDs-2^b^	0.17	0.41	0.47	0.64	0.28
CQDs-3^c^	1.33	1.65	4.00	3.54	1.92

a CQDs-1: GNP + glucose.

b CQDs-2: GNP + extracted gardenia seeds.

c CQDs-3: GNP + gardenia seeds.

d ND.

To further investigate the surface functionalities, X-ray photoelectron spectroscopy (XPS) was utilized. The full XPS spectra of all CQDs possessed Na_2p_, Na_2s_, C_1s_, Na_KLL_, O_1s_, O_KLL_, and Na_1s_ signals (Figure S3a). Figure S3b,c for all CQDs revealed distinct chemical environments for C_1s_ and O_1s_, respectively. The chemical environment and peak area are listed in Table 3. All CQDs exhibit higher contents of C=C and *O–(C=O) signals, suggesting that these components might effectively facilitate electron movement, representing a potential luminescence mechanism.

**Table 3:** Chemical Environments and Peak Ratios of C_1s_ and O_1s_ for the Prepared CQDs

**chemical environment and peak ratios**	**functional group**	**binding energy (eV)**	**CQDs-1**	**CQDs-2**	**CQDs-3**
C_1S_	C=C	284.5	33.6%	75.0%	95.2%
	C–C, C–H	285.0	33.4%	21.6%	0%
	C–OH, C–O–C	286.5	19.2%	2.7%	0%
	C=O	288.0	8.1%	0.6%	2.3%
	O–C=O	289.0	5.7%	0%	2.5%
O_1s_	C=O	531.0	56.4%	53.5%	51.5%
	O–(C=O*)–C	531.6	26.8%	31.2%	29.2%
	C–OH	533.6	0.3%	0.6%	0.3%
	*O–(C=O)	534.8	16.5%	14.6%	19.0%

### 3.2 Antibacterial Evaluation and Bacterial-CQD Conjugation

To evaluate their cytotoxicity, we conducted a comprehensive study examining the impact of CQDs on the viability and inhibition zone of *E. coli* (Figure S4) while comparing to the control experiment using benzoic acid. The results indicated that the synthesized CQDs did not impede the growth of *E. coli*, implying their compatibility with bacterial cells. Therefore, CQDs offer promising alternatives to traditional organic dyes and semiconductor quantum dots, highlighting their potential as probes for imaging various types of bacteria.

**Figure 2:**
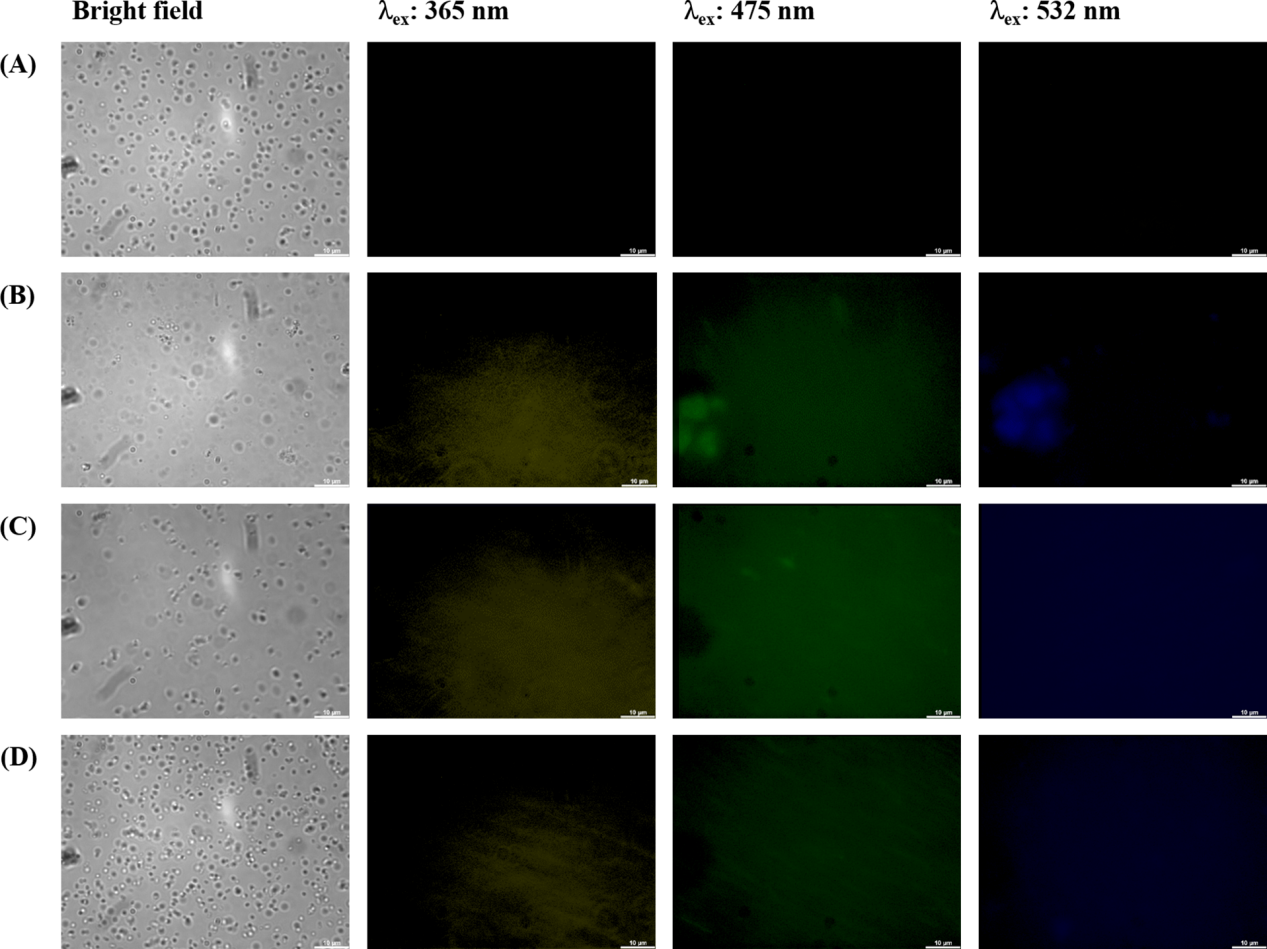
Confocal laser microscopic images of *E. coli* (A) without and with (B) CQDs-1 (50 mg GNP + 50 mg glucose), (C) CQDs-2 (50 mg GNP + 50 mg extracted gardenia seed), and (D) CQDs-3 (50 mg GNP + 50 mg gardenia seed) prepared at 220 °C (25.6 mg/mL) after incubation at 37 °C for 6 h: bright-field and fluorescence mode at excitation wavelengths of 365, 475, and 532 nm.

Figure 2 illustrates PL images of CQDs-labeled bacterial (*E. coli*) cells. Laser excitations at 365, 475, and 532 nm were employed to activate the internalized CQDs, resulting in PL emissions in three distinct colors: blue, green, and yellow. The illuminated regions precisely corresponded to the cell locations, indicating the uptake and retention of CQDs within the cells. It is crucial to emphasize that the specific transport mechanisms of CQDs across the bacterial cell wall can vary with size, surface properties, and composition of the carbon dots as well as the bacterial strain and other environmental conditions. From our observation, small-sized and highly water-soluble CQDs may passively diffuse through the pores and channels present in the bacterial cell wall. In addition, CQDs may be actively taken up by bacteria through endocytosis or other energy-dependent processes. In this mechanism, the CQDs might bind to cell surface receptors first before they can be transported into the cell.^10^ As depicted in Figure 2A, bacteria without CQDs did not exhibit any PL emission, while bacteria with CQDs showed clear PL signal uniformly throughout the cells, confirming the facile passage of CQDs through the cell membranes and their subsequent uptake by the cells. These observations strongly suggest the accumulation of the synthesized CQDs in both the cell membranes and cytoplasm.

### 3.3 Preparation and Characterization of AuNPs Using CQDs as a Reducing Agent and a Stabilizer

**Figure 3:**
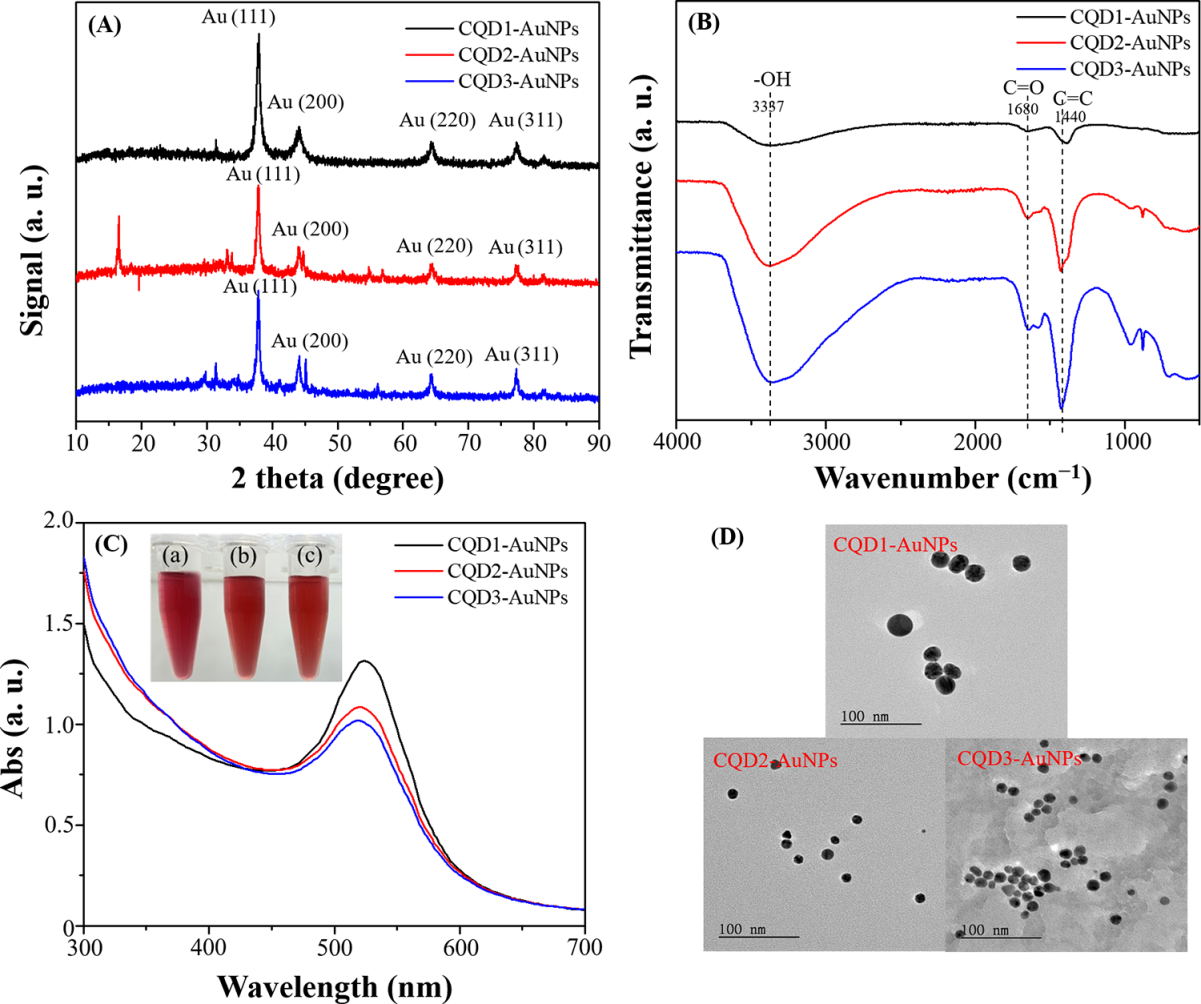
(A) XRD patterns, (B) FT-IR spectra, (C) UV–vis spectra (inset: photographic images of the prepared AuNP solutions. Photography courtesy of Xuan-Wei Fang. Copyright 2024), and (D) TEM images of AuNPs prepared by using (a) CQDs-1 (50 mg GNP + 50 mg glucose), (b) CQDs-2 (50 mg GNP + 50 mg extracted gardenia seed), and (c) CQDs-3 (50 mg GNP + 50 mg gardenia seed) as a reducing and protecting reagent.

In addition to cellular staining, this study investigated the dual roles of CQDs as both reducing agents and stabilizers in the reduction of chloroauric acid to produce CQDs-AuNPs. Figure 3A illustrates the XRD results, showing diffraction peaks at 2θ = 37.80°, 44.02°, 64.52°, and 77.60°, corresponding to crystallographic planes (111), (200), (220), and (311). The comparison of diffraction peaks confirmed that the CQDs-AuNPs synthesized through a one-pot method indeed possessed a face-centered cubic structure, consistent with the reference pattern (JCPDS No. 04–0784).^32−34^ During the synthesis process, the crystalline structure of CQDs-AuNPs becomes more pronounced, and due to the relatively lower concentration of CQDs, the XRD pattern predominantly displays the crystalline form of CQDs-AuNPs (Figure 3A). Before the introduction of Au^3+^ ions, the XRD analysis revealed a distinct peak for CQDs at 2θ = 22°, indicative of a graphite-like structure (Figure S1A).^28−31^Figure 3B presents the FT-IR analysis of CQD1-AuNPs, CQD2-AuNPs, and CQD3-AuNPs, utilizing CQDs-1, CQDs-2, and CQDs-3 as reducing agents and stabilizers, revealing the presence of −OH functional groups at 3337 cm^–1^. Additionally, vibrations associated with C=O and C=C were also observed at 1680 and 1440 cm^–1^, respectively. These functional groups originated from CQDs, indicating that CQDs not only served as reducing agents but also acted as stabilizing agents for the AuNPs. Among them, CQD2-AuNPs and CQD3-AuNPs exhibited stronger vibrational signals, indirectly suggesting a higher concentration of functional groups on the AuNP surface, preventing nanoparticle aggregation, and ensuring stability.

Figure 3C depicts the UV–vis spectra of prepared CQD1-AuNPs, CQD2-AuNPs, and CQD3-AuNPs. All three CQD-AuNPs exhibited distinct surface plasmon resonance characteristic peaks of AuNPs (λ_max, CQD1-AuNPs_: 524 nm, λ_max, CQD2-AuNPs_: 520 nm, λ_max, CQD3-AuNPs_: 520 nm). Additionally, we attempted the synthesis of AuNPs using gardenia seeds (both those that had undergone extraction and those in their natural state) and pure GNP. The results are illustrated in Figure S5. The surface plasmon resonance characteristic peaks of AuNPs in all three cases lacked distinct features, showing that gardenia seeds and pure GNP are less effective in the synthesis of AuNPs. Finally, TEM analysis confirmed the spherical morphology of CQD1-AuNPs, CQD2-AuNPs, and CQD3-AuNPs, with sizes of 24.1 ± 3.8, 11.8 ± 0.5, and 12.0 ± 0.3 nm, respectively (Figure 3D).

The full XPS spectra of all CQDs-AuNPs exhibited signals for Au_4f_, C_1s_, Na_KLL_, O_1s_, O_KLL_, and Na_1s_ as shown in Figure S6a. The atomic percentage ratio of C:O:Au for CQD1-AuNPs was 62.4:36.4:1.3, for CQD2-AuNPs was 69.4:30.6:<0.1, and for CQD3-AuNPs was 67.1:32.8:<0.1. Figure S6b–d provides detailed chemical environments for C_1s_, O_1s_, and Au_4f_, respectively. Comparing the peak intensities in the spectra, CQD2-AuNPs and CQD3-AuNPs exhibited larger C 1s peaks, indicating a higher concentration of functional groups on the CQDs-AuNPs, consistent with the FT-IR results. The Au_4f_ core-level spectra of the three sets of CQDs-AuNPs revealed features related to Au^0^, Au^+^, and Au^3+^ (Figure S6d). Specifically, the Au^0^ component showed peaks at Au_4f7/2_ (84.0 eV) and Au_4f5/2_ (87.7 eV). The Au^+^ components included peaks at Au_4f7/2_^+^ (85.6 eV) and Au_4f5/2_^+^ (89.3 eV), while the Au^3+^ components showed peaks at Au_4f7/2_^3+^ (87.3 eV) and Au_4f5/2_^3+^ (90.9 eV). The chemical environments and peak areas of C_1s_, O_1s_, and Au_4f_ are summarized in Table 4. In Table 4, the C_1s_ and O_1s_ compositions indicate that the surface of the synthesized CQDs-AuNPs is predominantly composed of C=C and C=O functional groups. Regarding the oxidation states in the Au_4f_ composition, all CQDs-AuNPs exhibit Au^0^ as the main state. However, in comparison to CQD2-AuNPs and CQD3-AuNPs, CQD1-AuNPs show a higher content of the Au^3+^ oxidation state, suggesting that the reducing power of CQDs-1 is weaker than that of CQDs-2 and CQDs-3.

**Table 4:** Chemical Environments and Peak Ratios of C_1s_, O_1s_, and Au_4f_ for the Prepared CQDs-AuNPs

**chemical environment and peak ratios**	**functional group**	**binding energy(eV)**	**CQD1-AuNPs**	**CQD2-AuNPs**	**CQD3-AuNPs**
C_1S_	C=C	284.5	75.6%	75.0%	74.4%
	C–C, C–H	285.0	4.8%	21.6%	12.4%
	C–OH, C–O–C	286.5	2.6%	2.7%	0%
	C=O	288.0	7.5%	0.6%	1.6%
	O–C=O	289.0	9.5%	0%	11.6%
O_1s_	C=O	531.0	57.8%	48.0%	43.7%
	O–(C=O*)–C	531.6	20.8%	35.4%	40.2%
	C–OH	533.6	0.3%	0.3%	0.2%
	*O–(C=O)	534.8	21.2%	16.3%	15.9%
Au_4f_	Au 4f_7/2_^0^	84.0	62.6%	67.5%	54.7%
	Au 4f_7/2_^+^	85.6	2.0%	6.3%	20.8%
	Au 4f_7/2_^+3^	87.3	34.0%	3.9%	15.3%
	Au 4f_5/2_^0^	87.7	0%	6.3%	0%
	Au 4f_5/2_^+^	89.3	1.3%	10.8%	7.1%
	Au 4f_5/2_^+3^	90.9	0.1%	5.2%	2.1%

In addition, the FT-IR and XPS analyses in the study substantiate that CQDs’ functional groups serve as both reducing agents and stabilizers for AuNPs. FT-IR analysis highlighted crucial surface functional groups on CQDs, like hydroxyl, carbonyl, and carboxylic acid groups, essential for their reducing activity (Figure S1B). Concurrently, XPS analysis offered insights into the chemical composition, revealing that the content of partial oxygen components in different oxidation states in CQDs-AuNPs was lower compared to CQDs alone, suggesting the involvement of hydroxyl and carbonyl groups in AuNPs’ synthesis and stability (Tables 3 and 4 and Figure 3B). These findings collectively affirm CQDs’ dual role in the ecofriendly production of AuNPs, showcasing their capacity to not only convert gold ions to nanoparticles but also to ensure their stability and prevent aggregation.

### 3.4 Catalytic Performance of NaBH_4_ Reduction of 4-NP in the Presence of CQDs-AuNPs

**Figure 4:**
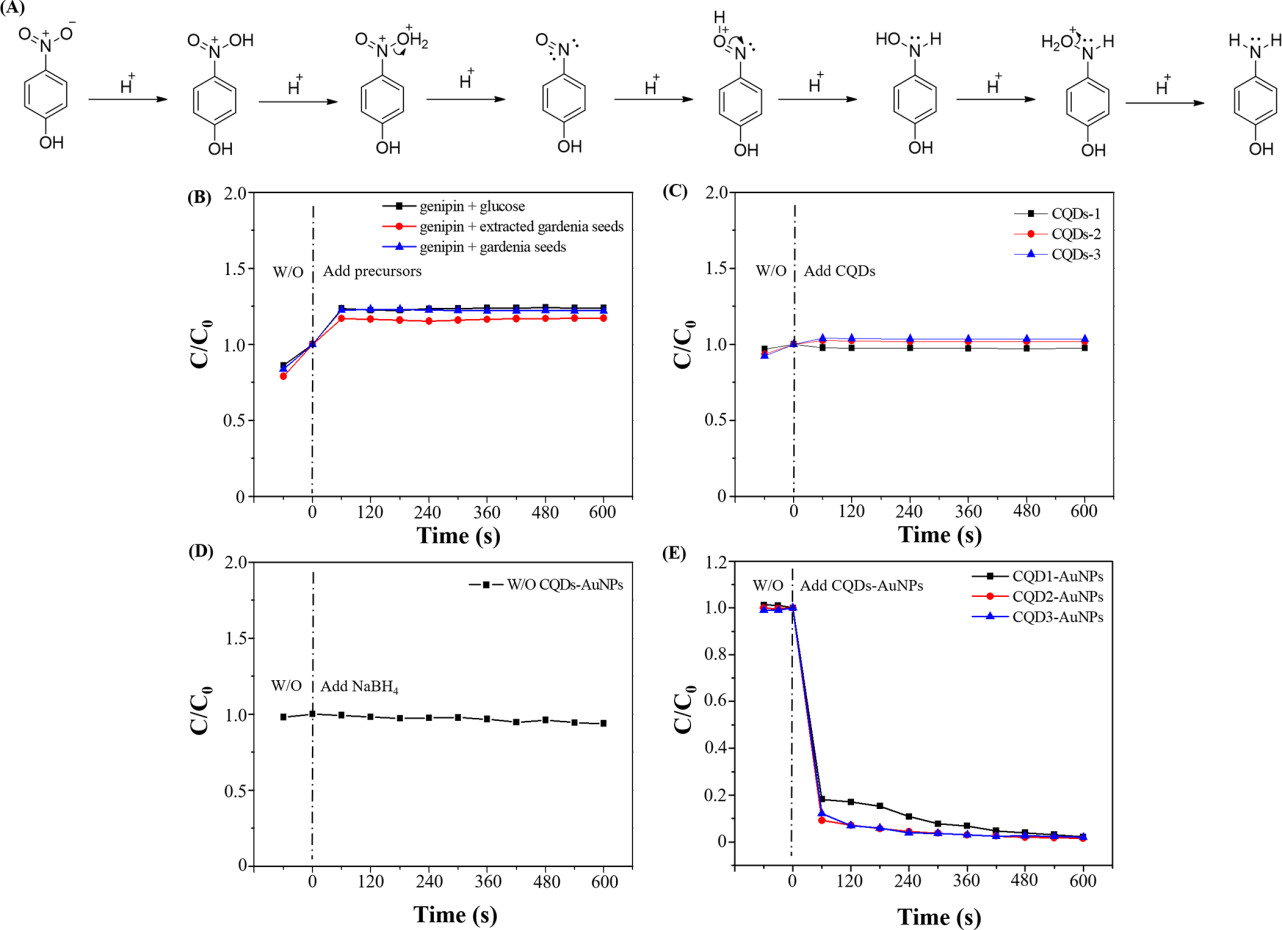
(A) Scheme of NaBH_4_ reduction of 4-NP to 4-AP, (B) catalytic performances of reduction of 4-NP in the presence of (B) NaBH_4_/CQDs precursors, (C) NaBH_4_/CQDs, (C) NaBH_4_, and (D) NaBH_4_/CQDs-AuNPs.

The prepared CQDs-AuNPs were employed to catalyze the reduction of 4-NP. Figure 4A illustrates a plausible mechanism for the catalytic reduction of 4-NP. The stepwise hydrogenation process facilitates the reduction of the nitro group of 4-NP to an amino group. However, in the absence of NaBH_4_ and CQDs-AuNPs, the catalytic reaction did not occur spontaneously. Without NaBH_4_, the adsorption of H^+^ to the nitro group is challenging. The addition of NaBH_4_ disrupts the initial equilibrium, introducing a significant amount of H^+^. Nevertheless, in the absence of CQDs-AuNPs, effective catalysis of the reduction of 4-NP remains unattainable due to the substantial energy barrier in the reaction process. Thus, H^+^ ions are adsorbed onto the surface of CQDs-AuNPs, constituting a pivotal step in the catalytic reaction. The introduction of CQDs-AuNPs lowers the energy barrier, expediting the reaction kinetics and providing surfaces for the adsorption of H^+^, thereby achieving the catalytic reduction effect.^25^

To validate the pivotal role of CQDs-AuNPs in the catalytic reduction of 4-NP, a comprehensive series of experiments was conducted. The study monitored the characteristic absorption peak of ionized 4-NP at 400 nm, assessing catalytic reduction by observing peak changes and calculating the *C*/*C*_0_ values (where *C*_0_ is the initial concentration of ionized 4-NP without any additives and *C* is the concentration of ionized 4-NP at time after the addition of additives). Figure 4B details catalysis experiments using the precursors of the three sets of CQDs. The results indicate that experiments employing only CQD precursor materials were ineffective in catalyzing the reduction of 4-NP. No significant reduction of 4-NP was observed during the 10 min monitoring period after the addition of precursors. Figure 4C outlines experiments using the prepared CQDs for catalytic reduction. As depicted in Figure 4C, the three sets of CQDs also failed to catalyze the reduction of 4-NP successfully. In Figure 4D, a quartz cuvette was treated with NaBH_4_ and 4-NP without adding CQDs-AuNPs. The experiment exhibited an absorption characteristic peak at 317 nm, indicating the presence of 4-NP. After the addition of NaBH_4_, the absorption peak shifted from 317 to 400 nm, signifying the ionization of 4-NP and a color change from colorless to bright yellow. Continuous monitoring for 10 min did not effectively reduce the ionized 4-NP to 4-AP, as the absorption values of the characteristic peak at 400 nm did not significantly decrease. Upon adding CQDs-AuNPs, catalytic reduction reactions were initiated, converting ionized 4-NP to 4-AP. Consequently, the solution transitioned from bright yellow to colorless and a characteristic peak of 4-AP at 300 nm was observed, indicating the successful reduction of 4-NP. As illustrated in Figure 4E, with the addition of CQDs-AuNPs, 99.7% of 4-NP was effectively reduced to 4-AP within 10 min.

Subsequently, taking CQD3-AuNPs as an example, optimization was conducted for NaBH_4_ concentration and CQD3-AuNPs content, as shown in Figure S7. When NaBH_4_ concentrations varied from 9 to 45 mM in the presence of CQD3-AuNPs, there is strong evidence that 99.7% 4-NP was reduced to 4-AP within a 10 min reaction time (Figure S7A). To provide a sufficient H^+^ concentration, 36 mM NaBH_4_ was selected as the optimized concentration. Next, optimization of CQD3-AuNPs content was performed, and experimental results as shown in Figure S7B indicated that a CQD3-AuNP content of 9% achieves a 99.7% reduction activity of 4-NP, making it the optimal addition amount for CQD3-AuNPs. Under these optimized conditions, the catalytic reduction reaction followed a pseudo-first-order kinetic behavior with kinetic constants of 0.07, 0.11, and 0.10 s^–1^ for CQD1-AuNPs, CQD2-AuNPs, and CQD3-AuNPs, respectively.

### 3.5 Long-Term Stability of Catalytic Activity and Applications of CQD3-AuNPs

**Figure 5:**
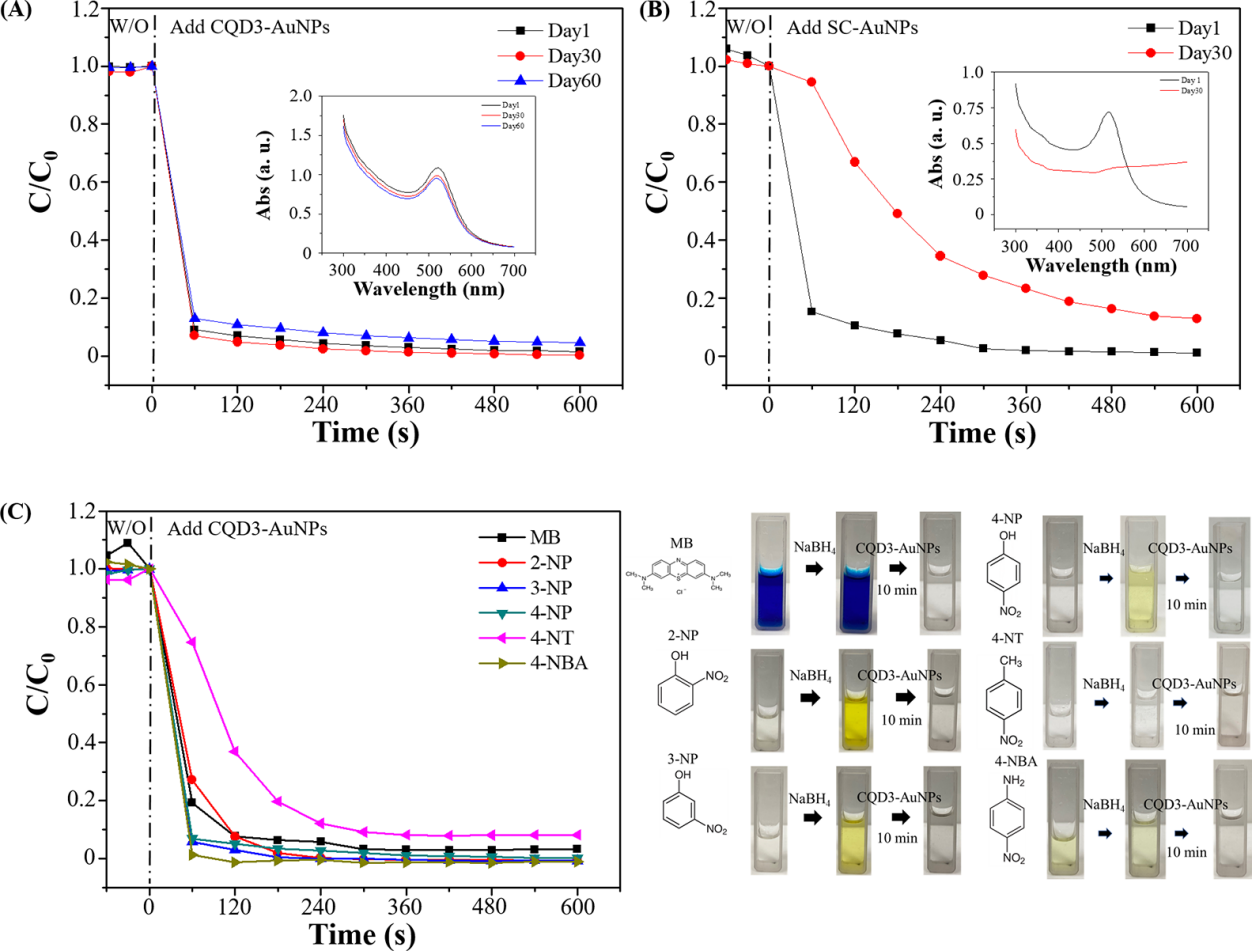
Long-term NaBH_4_ catalytic performance of reduction of 4-NP by (A) CQD3-AuNPs and (B) SC-AuNPs. Inset: UV–vis spectra of AuNPs at different storage times. (C) Catalytic performances and photograph images of the reduction of aromatic nitro compounds by CQD3-AuNPs in the presence of NaBH_4_ (photography courtesy of Xuan-Wei Fang. Copyright 2024).

Long-term storage of CQDs-AuNP ensures potential practical applications. The catalytic reduction stability of CQD3-AuNPs in the presence of NaBH_4_ was tested as shown in Figure 5A. On the freshly prepared CQD3-AuNPs, the catalytic reduction efficiency reached 99.7% within 10 min. After 60 days of storage, it still maintained a high catalytic efficiency, reducing 99.6% of 4-NP to 4-AP within 10 min. In comparison, gold nanoparticles synthesized by sodium citrate (SC-AuNPs) showed a decrease in catalytic reduction efficiency from 99.9% to 89.2% after 30 days, suggesting a potential decrease in the stability and aggregation of SC-AuNPs (Figure 5B). Therefore, it can be concluded that CQD3-AuNPs not only exhibit excellent stability, preventing aggregation, but also demonstrate outstanding catalytic efficiency in catalytic reduction experiments. These results highlight that CQD3-AuNPs possess excellent stability and catalytic performance, making them a promising material for catalytic reactions.

Under the optimized catalytic conditions, catalytic reduction experiments were conducted not only for 4-NP but also for various aromatic nitro compounds to further understand the efficacy of CQD3-AuNPs in the presence of NaBH_4_ in a broader range of pollutant applications. These compounds include methylene blue (MB), 2-nitrophenol (2-NP), 3-nitrophenol (3-NP), 4-nitrotoluene (4-NT), and 4-nitrobenzenamine (4-NBA). The disappearance of characteristic peaks and the calculation of the C/C_0_ ratio were employed to assess the extent of catalytic reduction. After each aromatic nitro compound was allowed to equilibrate for 60 s at room temperature, CQD3-AuNPs were added and mixed with constant stirring for 10 min. The concentration of nitro compounds was evaluated to determine the degree of catalytic reduction. According to the results in Figure 5C, the 10 min reaction achieved 96.9% reduction for MB (color change from blue to colorless), 100% reduction for 2-NP (color change from yellow to colorless), 100% reduction for 3-NP (color change from yellow to colorless), 91.9% reduction for 4-NT, and 100% reduction for 4-NBA (color change from light yellow to colorless) catalyzed by CQD3-AuNPs in the presence of NaBH_4_. The reaction times were reduced from 10 to 1 min to compare the performance of different nitro compounds. After 1 min of reduction, it was observed that MB, 4-NP, and 4-NT exhibited comparatively lower catalytic efficiency than 2-NP, 3-NP, and 4-NBA. This discrepancy may be attributed to factors such as molecular structure, types of functional groups, and their positions, which can influence the catalytic process. In summary, CQD3-AuNPs demonstrated effective catalytic reduction not only on 4-NP but also on various aromatic nitro compounds, showcasing excellent wastewater remediation applications. The catalytic reduction efficiencies for all compounds exceeded 90%, demonstrating promising catalytic performance and application prospects.

## 4 Conclusions

Utilizing a pyrolysis method at 220 °C, this study synthesized three variants of CQDs using GNP and different organic sources, leading to the creation of CQDs-1, CQDs-2, and, notably, CQDs-3 with the highest quantum yield of 4.0%. Through rigorous characterization (XRD, FTIR, TEM, and XPS), the structural integrity and nanoscale dimensions of these CQDs were confirmed, alongside their excellent biocompatibility and potent reducing capabilities for synthesizing AuNPs. The resultant CQD3-AuNPs stood out for their exceptional stability and catalytic efficiency, achieving up to 99.7% reduction of various aromatic nitro compounds within just 10 min and maintaining nearly unchanged efficiency after 60 days. This work not only demonstrates a straightforward, scalable, and waste-minimizing method for producing CQDs and CQD-stabilized AuNPs but also highlights their promising applications in bacterial imaging and wastewater treatment, setting a precedent for future advancements in nanotechnology and environmental science.

## Supporting Information Available

Figure S1: XRD, FT-IR spectra, and TEM images of CQDs; Figure S2: excitation-dependent emission spectra of CQDs; Figure S3: full-range XPS and high-resolution XPS spectrum of CQDs; Figure S4: photograph images of the inhibition zone of CQDs; Figure S5: UV–vis spectra of AuNPs; Figure S6: full-range XPS and high-resolution XPS spectrum of CQDs-AuNPs; Figure S7: optimum evaluations of reduction of 4-NP in the presence of CQD3-AuNPs (DOCX)

## References

[ref1] Mansuriya B. D.; Altintas Z. Carbon Dots: Classification, properties, synthesis, characterization, and applications in health care—An updated review (2018–2021). Nanomaterials 2021, 11 (10), 2525 10.3390/nano11102525. 34684966 PMC8541690

[ref2] Wei S.-C.; Lin Y.-W.; Chang H.-T. Carbon dots as artificial peroxidases for analytical applications. J. Food Drug Anal. 2020, 28 (4), 558 10.38212/2224-6614.1090. 35696142 PMC9261811

[ref3] Chu H.-W.; Unnikrishnan B.; Anand A.; Lin Y.-W.; Huang C.-C. Carbon quantum dots for the detection of antibiotics and pesticides. J. Food Drug Anal. 2020, 28 (4), 539 10.38212/2224-6614.1269. 35696146 PMC9261805

[ref4] Yang C.-R.; Lin Y.-S.; Wu R.-S.; Lin C.-J.; Chu H.-W.; Huang C.-C.; Anand A.; Unnikrishnan B.; Chang H.-T. Dual-emissive carbonized polymer dots for the ratiometric fluorescence imaging of singlet oxygen in living cells. J. Colloid Interface Sci. 2023, 634, 575–585. 10.1016/j.jcis.2022.12.076. 36549206

[ref5] Barve K.; Singh U.; Yadav P.; Bhatia D. Carbon-based designer and programmable fluorescent quantum dots for targeted biological and biomedical applications. Mater. Chem. Front. 2023, 7 (9), 1781–1802. 10.1039/D2QM01287A.

[ref6] Lin X.; Xiong M.; Zhang J.; He C.; Ma X.; Zhang H.; Kuang Y.; Yang M.; Huang Q. Carbon dots based on natural resources: Synthesis and applications in sensors. Microchem J. 2021, 160, 105604 10.1016/j.microc.2020.105604.

[ref7] Xu D.; Lin Q.; Chang H.-T. Recent advances and sensing applications of carbon dots. Small Methods 2020, 4 (4), 1900387 10.1002/smtd.201900387.

[ref8] Liu J.; Li R.; Yang B. Carbon dots: A new type of carbon-based nanomaterial with wide applications. ACS Central Sci. 2020, 6 (12), 2179–2195. 10.1021/acscentsci.0c01306. PMC7760469 33376780

[ref9] Chahal S.; Macairan J.-R.; Yousefi N.; Tufenkji N.; Naccache R. Green synthesis of carbon dots and their applications. RSC Adv. 2021, 11 (41), 25354–25363. 10.1039/D1RA04718C. 35478913 PMC9037072

[ref10] Tsai H.-W.; Wu T.; Hsieh C.-L.; Fu S.-F.; Wu M.-Y.; Lin Y.-W. Green synthesis of gardenia seeds-based carbon dots for bacterial imaging and antioxidant activity in aqueous and oil samples. RSC Adv. 2023, 13 (42), 29283–29290. 10.1039/D3RA06293G. 37809029 PMC10557051

[ref11] Guo S.; Zhang R.; Liu Y.; Zhang Q.; Liu X.; Wu X.; Li B. Synthesis, applications in therapeutics, and bioimaging of traditional Chinese medicine-derived carbon dots. Carbon Lett. 2023, 34, 545 10.1007/s42823-023-00615-y.

[ref12] Das R.; Bandyopadhyay R.; Pramanik P. Carbon quantum dots from natural resource: A review. Mater. Today Chem. 2018, 8, 96–109. 10.1016/j.mtchem.2018.03.003.

[ref13] Sachdev A.; Gopinath P. Green synthesis of multifunctional carbon dots from coriander leaves and their potential application as antioxidants, sensors and bioimaging agents. Analyst 2015, 140 (12), 4260–4269. 10.1039/C5AN00454C. 25927267

[ref14] Chiou Y.-R.; Lin C.-J.; Harroun S. G.; Chen Y.-R.; Chang L.; Wu A.-T.; Chang F.-C.; Lin Y.-W.; Lin H.-J.; Anand A.; Unnikrishnan B.; Nain A.; Huang C.-C. Aminoglycoside-mimicking carbonized polymer dots for bacteremia treatment. Nanoscale 2022, 14 (32), 11719–11730. 10.1039/D2NR01959K. 35913451

[ref15] Skolariki T. A.; Chatzimitakos T. G.; Sygellou L.; Stalikas C. D. Two-birds-with-one-stone synthesis of hydrophilic and hydrophobic fluorescent carbon nanodots from Dunaliella salina biomass as 4-nitrophenol nanoprobes based on inner filter effect and first derivative redshift of emission band. Nanomaterials 2023, 13 (10), 1689 10.3390/nano13101689. 37242105 PMC10223520

[ref16] Balakrishnan A.; Gaware G. J.; Chinthala M. Heterojunction photocatalysts for the removal of nitrophenol: A systematic review. Chemosphere 2023, 310, 136853 10.1016/j.chemosphere.2022.136853. 36243095

[ref17] Mishra S. R.; Gadore V.; Ahmaruzzaman M. Inorganic–organic hybrid quantum dots for AOP-mediated photodegradation of ofloxacin and para-nitrophenol in diverse water matrices. NPJ. Clean Water 2023, 6 (1), 78 10.1038/s41545-023-00291-5.

[ref18] Ma Z.; Li Y.; Lu Z.; Pan J.; Li M. A novel biosensor-based method for the detection of p-nitrophenol in agricultural soil. Chemosphere 2023, 313, 137306 10.1016/j.chemosphere.2022.137306. 36410515

[ref19] Yu W.; Qiu L.; Zhu J.; Chen S.; Song S. Fe-embedded ZIF-derived N-doped carbon nanoparticles for enhanced selective reduction of p-nitrophenol. J. Environ. Chem. Eng. 2023, 11 (2), 109609 10.1016/j.jece.2023.109609.

[ref20] Peng Y.; Bian Z.; Wang F.; Li S.; Xu S.; Wang H. Electrocatalytic degradation of p-nitrophenol on metal-free cathode: Superoxide radical (O_2_•^–^) production via molecular oxygen activation. J. Hazard. Mater. 2024, 462, 132797 10.1016/j.jhazmat.2023.132797. 37865078

[ref21] Abd El-Monaem E. M.; Eltaweil A. S.; El-Subruiti G. M.; Mohy-Eldin M. S.; Omer A. M. Adsorption of nitrophenol onto a novel Fe_3_O_4_-κ-carrageenan/MIL-125 (Ti) composite: process optimization, isotherms, kinetics, and mechanism. Environ. Sci. Pollut. Res. 2023, 30 (17), 49301–49313. 10.1007/s11356-023-25678-2. PMC10104928 36773266

[ref22] Feng A.; Lin C.; Zhou H.; Jin W.; Hu Y.; Li D.; Li Q. Catalytic transformation of 4-nitrophenol into 4-aminophenol over ZnO nanowire array-decorated Cu nanoparticles. Green Chem. Eng. 2023, 5, 205 10.1016/j.gce.2023.03.003.

[ref23] Akti F. Silver and zirconium mono-and bi-metallic silica mesoporous functionalized PDA materials as highly efficient reusable catalyst for reduction of 4-NP to 4-AP. Mater. Chem. Phys. 2023, 304, 127858 10.1016/j.matchemphys.2023.127858.

[ref24] Migdadi A.; Al-Bataineh Q. M.; Ahmad A. A.; Al-Khateeb H.; Telfah A. Titanium dioxide/reduced graphene oxide nanocomposites as effective photocatalytic for hazardous 4-nitrophenol. J. Alloy. Compd. 2024, 971, 172794 10.1016/j.jallcom.2023.172794.

[ref25] Ma A.; Yang W.; Gao K.; Tang J. Concave gold nano-arrows (AuCNAs) for efficient catalytic reduction of 4-nitrophenol. Chemosphere 2023, 310, 136800 10.1016/j.chemosphere.2022.136800. 36244421

[ref26] Hua Y.; Zhang J.; Zhang T.; Zhu A.; Xia G.; Zhang X.; Di L. Plasma synthesis of graphite oxide supported PdNi catalysts with enhanced catalytic activity and stability for 4-nitrophenol reduction. Catal. Today 2023, 418, 114069 10.1016/j.cattod.2023.114069.

[ref27] Das T. K.; Ghosh S. K.; Das N. C. Green synthesis of a reduced graphene oxide/silver nanoparticles-based catalyst for degradation of a wide range of organic pollutants. Nano Struc. Nano Obj. 2023, 34, 100960 10.1016/j.nanoso.2023.100960.

[ref28] Mao L.-H.; Tang W.-Q.; Deng Z.-Y.; Liu S.-S.; Wang C.-F.; Chen S. Facile access to white fluorescent carbon dots toward light-emitting devices. Ind. Eng. Chem. Res. 2014, 53 (15), 6417–6425. 10.1021/ie500602n.

[ref29] Li L.; Wu G.; Yang G.; Peng J.; Zhao J.; Zhu J.-J. Focusing on luminescent graphene quantum dots: current status and future perspectives. Nanoscale 2013, 5 (10), 4015–4039. 10.1039/c3nr33849e. 23579482

[ref30] Dong Y.; Pang H.; Yang H. B.; Guo C.; Shao J.; Chi Y.; Li C. M.; Yu T. Carbon-based dots co-doped with nitrogen and sulfur for high quantum yield and excitation-independent emission. Angew. Chem.-Int. Ed. 2013, 125 (30), 7954–7958. 10.1002/ange.201301114. 23761198

[ref31] Pan D.; Zhang J.; Li Z.; Wu M. Hydrothermal route for cutting graphene sheets into blue-luminescent graphene quantum dots. Adv. Mater. 2010, 22 (6), 734–738. 10.1002/adma.200902825. 20217780

[ref32] Tong C.; Tong X.; Cao Y.; Cai G.; Wang T.; Wei Q.; Shi S.; Guo Y. Solvent-mediated in situ growth and assembly of gold nanoparticles@ carbon dots for rapid colorimetric nonenzymatic alcohol sensing. Carbon 2022, 196, 154–162. 10.1016/j.carbon.2022.04.070.

[ref33] Chen W.; Shen J.; Chen S.; Yan J.; Zhang N.; Zheng K.; Liu X. Synthesis of graphene quantum dot-stabilized gold nanoparticles and their application. RSC Adv. 2019, 9 (37), 21215–21219. 10.1039/C9RA02758K. 35521309 PMC9066025

[ref34] Jaiswal A.; Gautam P. K.; Ghosh S. S.; Chattopadhyay A. Carbon dots mediated room-temperature synthesis of gold nanoparticles in poly(ethylene glycol). J. Nanopart. Res. 2014, 16, 1–14. 10.1007/s11051-013-2188-y.

