# Supplementary material for: Green Synthesis of Carbon Quantum Dots and Carbon Quantum Dot-Gold Nanoparticles for Applications in Bacterial Imaging and Catalytic Reduction of Aromatic Nitro Compounds
Source: ACS Omega. 2024 Apr 24;9(22):23573–83. doi: 10.1021/acsomega.4c00833 (PMC11154949; doi:10.1021/acsomega.4c00833)
Supplement: Supplementary file 1 — ao4c00833_si_001.docx [file ao4c00833_si_001.docx]

**Supporting information**

**Green synthesis of carbon quantum dots and carbon quantum dot-gold nanoparticles for applications in bacterial imaging and catalytic reduction of aromatic nitro compounds**

Xuan-Wei Fang^1^, Hao Chang^1^, Tsunghsueh Wu^2^, Chen-Hao Yeh ^3^, Fu-Li Hsiao^4^, Tsung-Shine Ko^5^, Chiu-Lan Hsieh^6^, Mei-Yao Wu ^7^, and Yang-Wei Lin^1, *^

^1^ Department of Chemistry, National Changhua University of Education, 1 Jin-De Road, Changhua City, 50007, Taiwan

^2^ Department of Chemistry, University of Wisconsin-Platteville, 1 University Plaza, Platteville, Wisconsin, 53818-3099, USA

^3^ Department of Materials Science and Engineering, Feng Chia University, 100, Wenhwa Road, Taichung City, 40724, Taiwan

^4^ Graduate Institute of Photonics, National Changhua University of Education, 1 Jin-De Road, Changhua City, 50007, Taiwan

^5^ Department of Electronic Engineering, National Changhua University of Education, 1 Jin-De Road, Changhua City, 50007, Taiwan

^6^ Department of Biology, National Changhua University of Education, 1 Jin-De Road, Changhua City, 50007, Taiwan

^7^ School of Post-baccalaureate Chinese Medicine, China Medical University, 91, Hsueh-Shih Road, Taichung, 40424, Taiwan

***** Correspondence: Department of Chemistry, National Changhua University of Education, Changhua City 50007, Taiwan; Tel: +886-4-7211190; E-mail: linywjerry@cc.ncue.edu.tw (Y.-W. Lin)


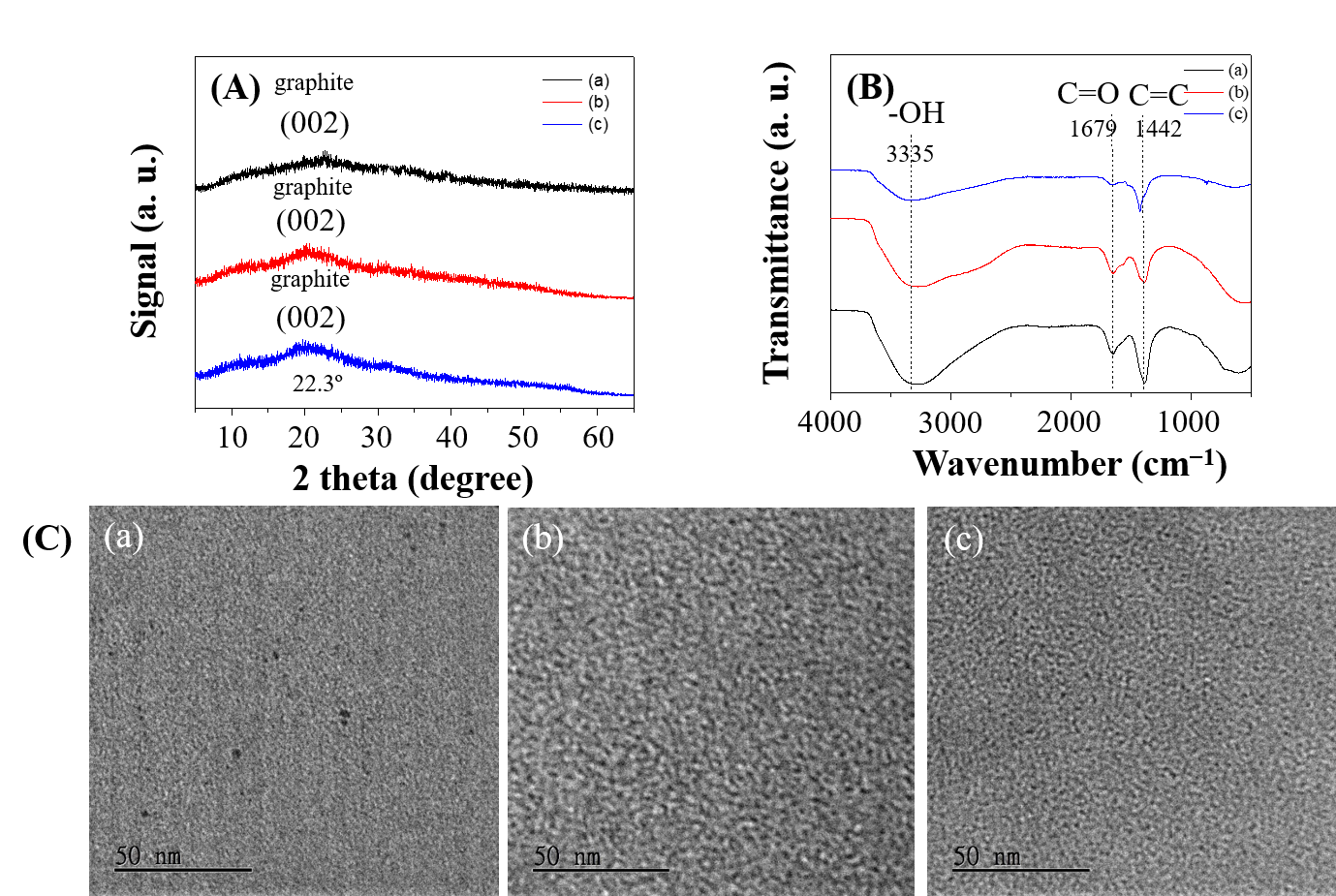


**Figure S1.** (A) XRD, (B) FT-IR spectra, and (C) TEM images of (a) CQDs-1 (50 mg GNP + 50 mg glucose), (b) CQDs-2 (50 mg GNP + 50 mg extracted gardenia seed), and (c) CQDs-3 (50 mg GNP + 50 mg gardenia seed) prepared at 220°C.


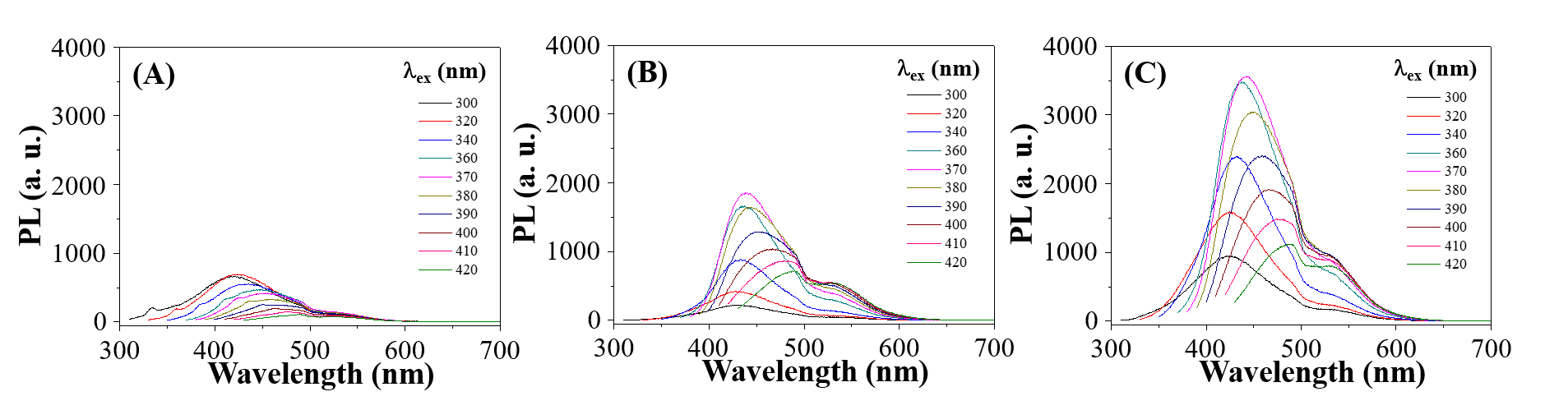


**Figure S2.** Excitation-dependent emissions spectra of (A) CQDs-1 (50 mg GNP + 50 mg glucose), (B) CQDs-2 (50 mg GNP + 50 mg extracted gardenia seed), and (C) CQDs-3 (50 mg GNP + 50 mg gardenia seed) prepared at 220°C.


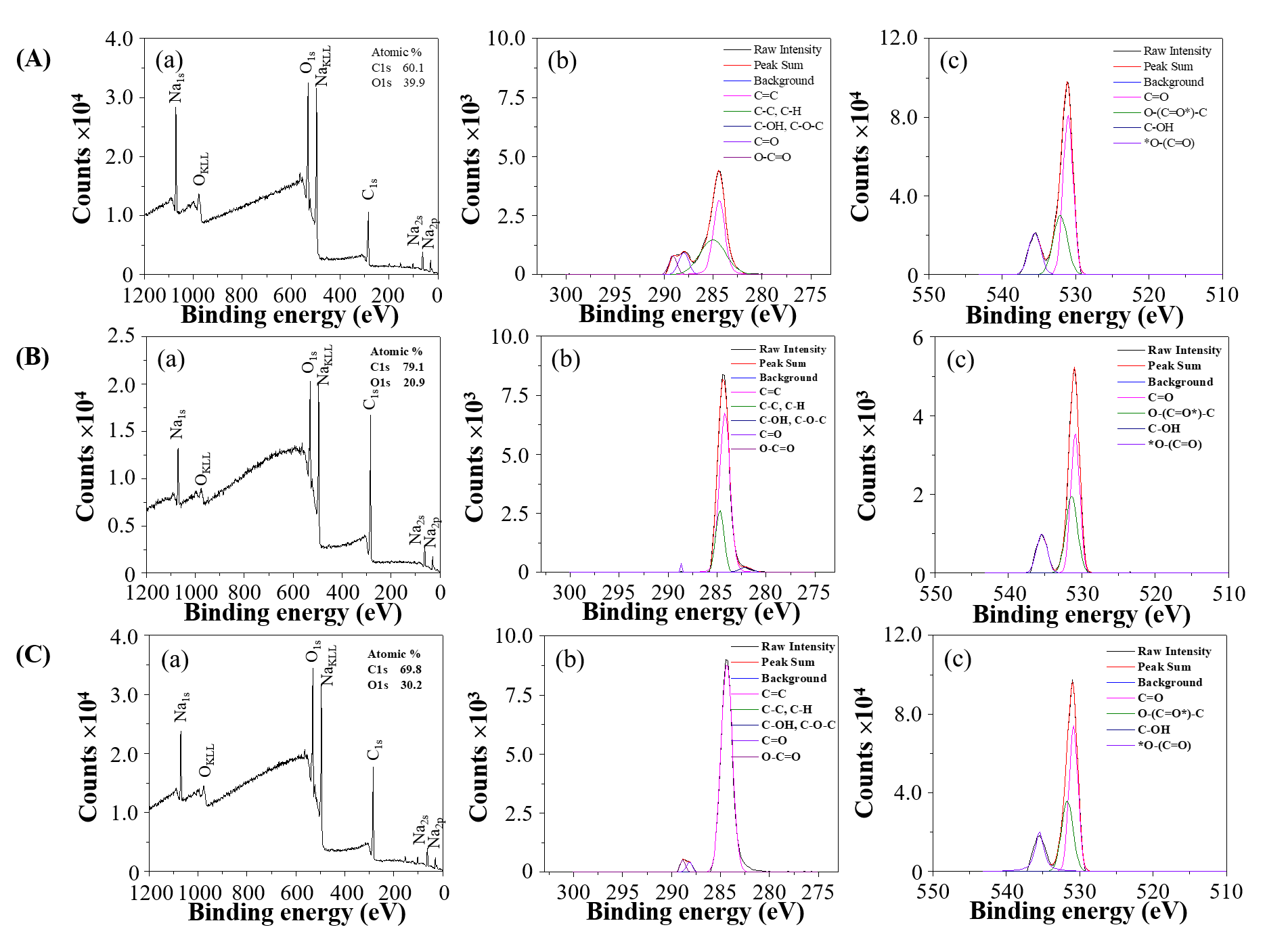


**Figure S3.** (a) Full-range XPS and high-resolution XPS spectrum of the (b) C1s and (c) O1s region of(A) CQDs-1 (50 mg GNP + 50 mg glucose), (B) CQDs-2 (50 mg GNP + 50 mg extracted gardenia seed), and (C) CQDs-3 (50 mg GNP + 50 mg gardenia seed) prepared at 220°C.


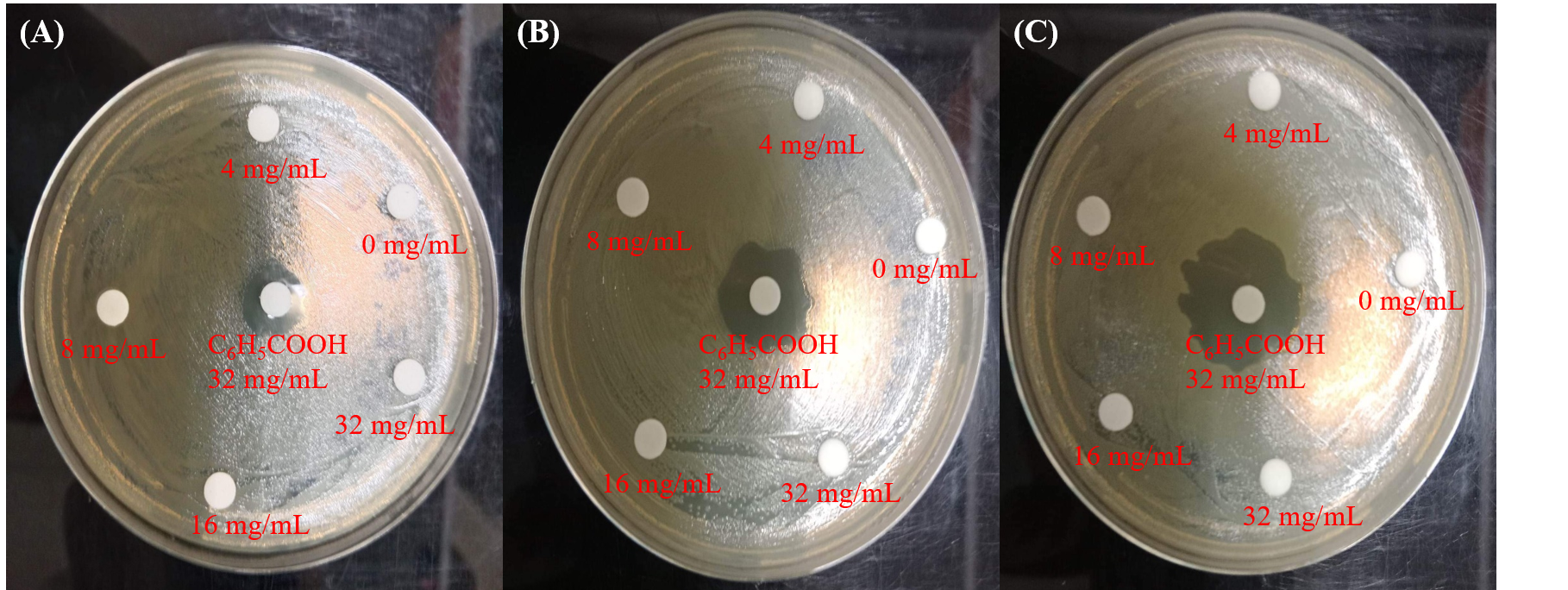


**Figure S4.** Photograph images of the inhibition zone of (A) CQDs-1 (50 mg GNP + 50 mg glucose), (B) CQDs-2 (50 mg GNP + 50 mg extracted gardenia seed), and (C) CQDs-3 (50 mg GNP + 50 mg gardenia seed) prepared at 220°C (0, 4, 8, 16, and 32 mg/mL) and BA (32 mg/mL) against *E. coli*. (Photography courtesy of Xuan-Wei Fang. Copyright 2024.)


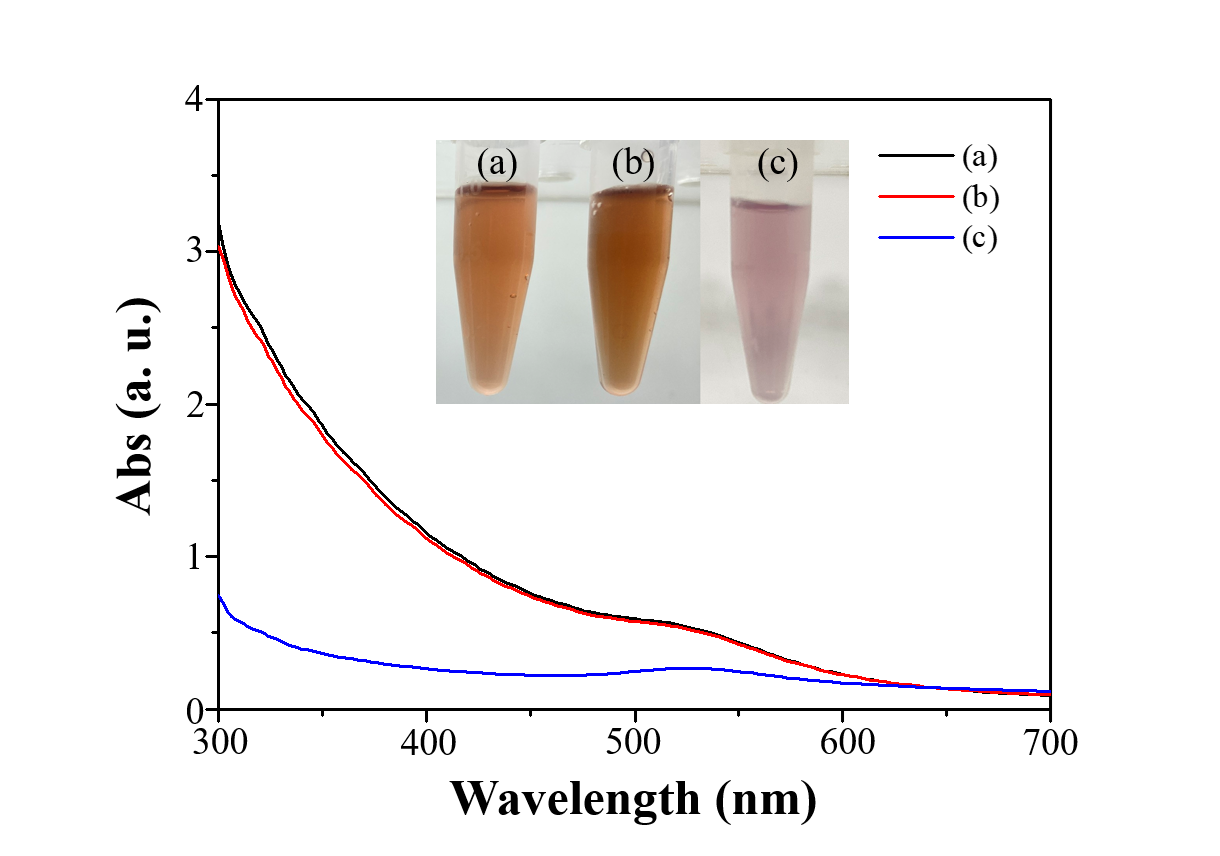


**Figure S5.** UV-Vis spectra of AuNPs prepared by (a) extracted gardenia seeds, (b) gardenia seeds, and (c) GNP. Inset: photograph images of the corresponding AuNP-solutions. (Photography courtesy of Xuan-Wei Fang. Copyright 2024.)


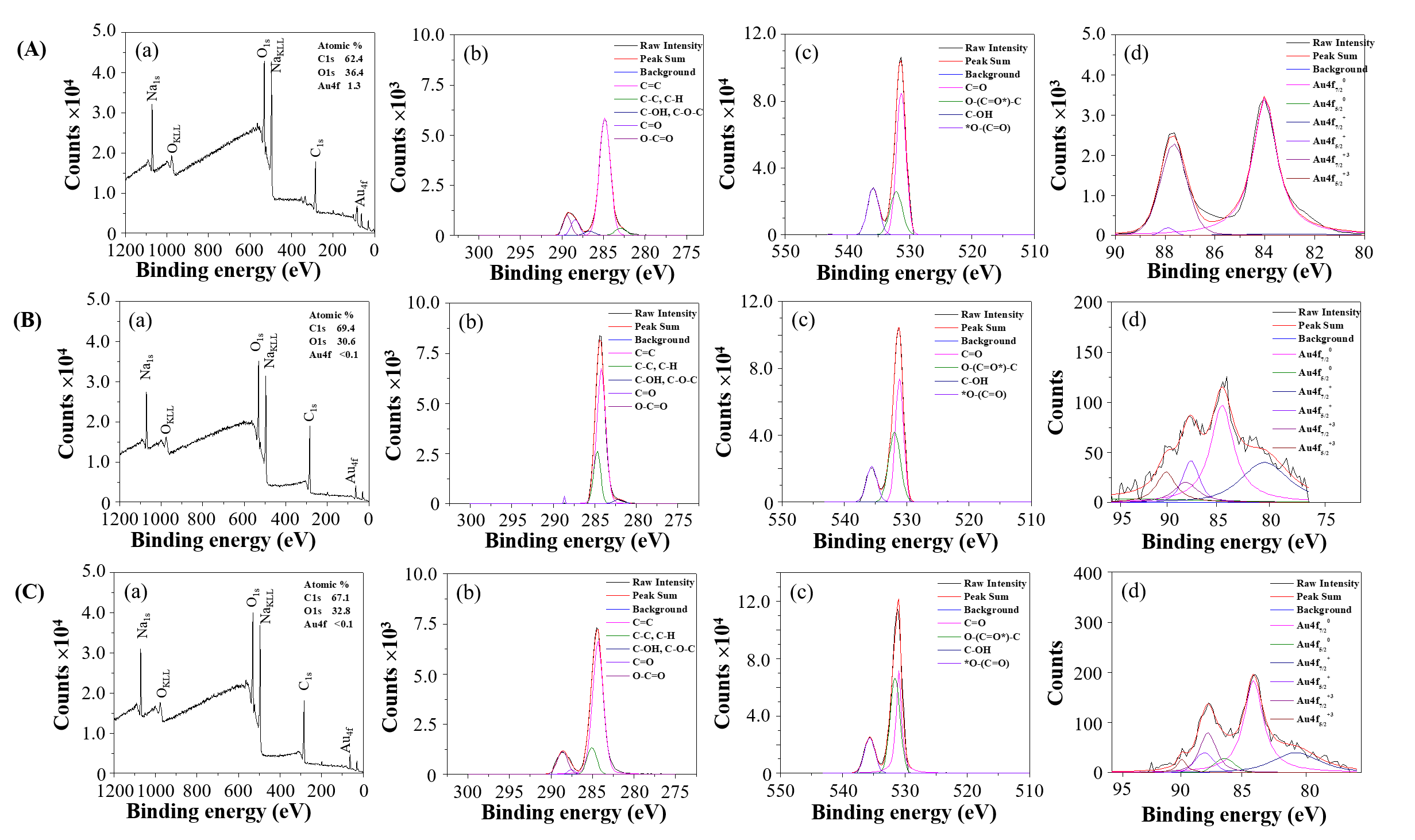


**Figure S6.** (a) Full-range XPS and high-resolution XPS spectrum of the (b) C1s, (c) O1s, and (d) Au4f region of (A) CQD1-AuNPs, (B) CQD2-AuNPs, and (C) CQD3-AuNPs prepared at 220°C.


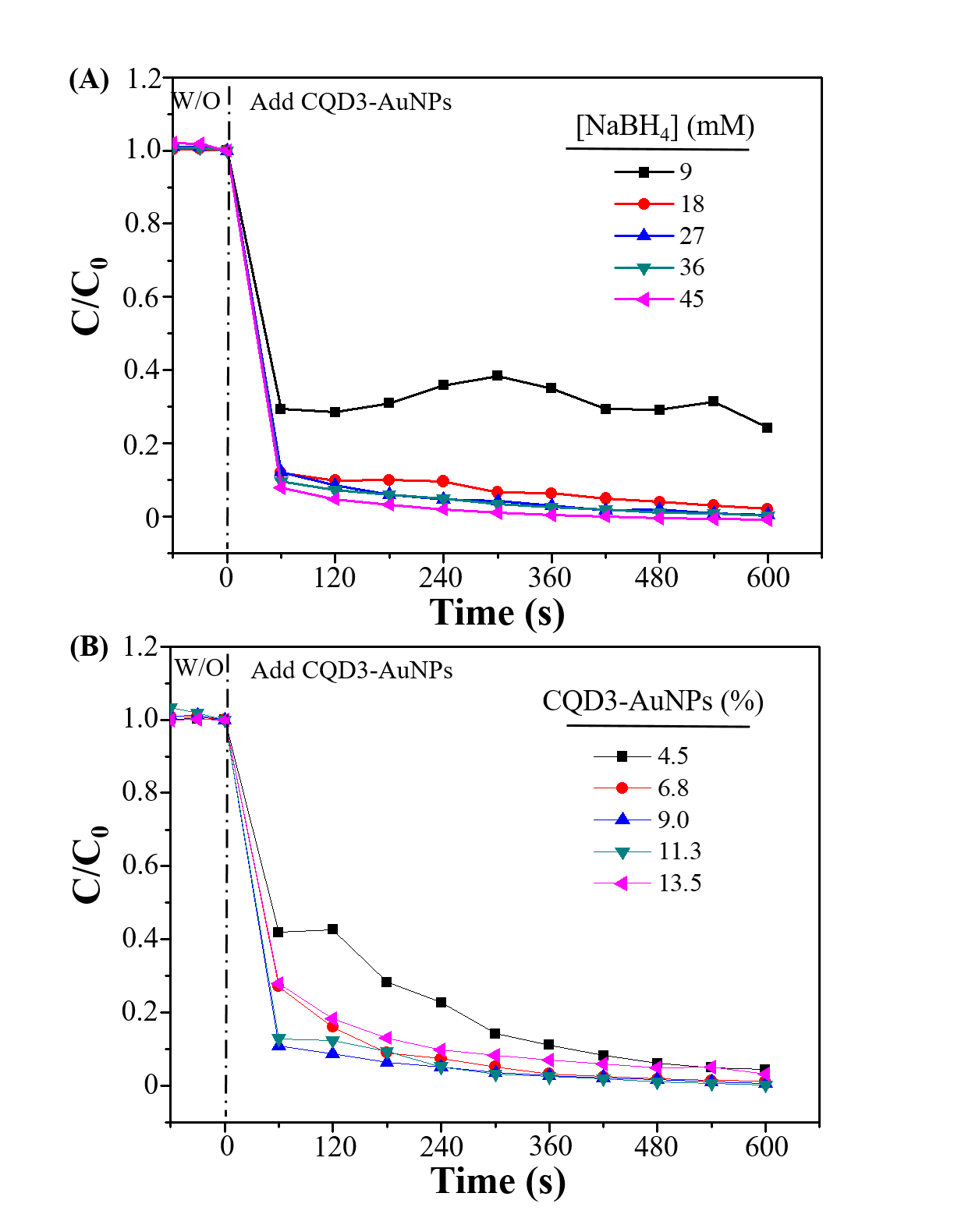


**Figure S7.** Optimum evaluations of NaBH_4_ reduction of 4-NP in the presence of CQD3-AuNPs: Effects of (A) NaBH_4_ concentration, and (B) CQD3-AuNPs volume.
